# Transforming non-muscle invasive bladder cancer (NMIBC) treatment: FDA approval of Anktiva in combination with BCG

**DOI:** 10.1097/MS9.0000000000002938

**Published:** 2025-01-31

**Authors:** Aimal Alam Khattak, Ahmed Javed, Prakriti Pokhrel, Sameen Mukhtar

**Affiliations:** a Department of Internal Medicine, Ayub Medical College, Abbottabad, Pakistan; b Kathmandu Medical College and Teaching Hospital, Kathmandu, Nepal; c Department of Internal Medicine, Dow University of Health Sciences, Karachi, Pakistan

## Background

Bladder cancer is a leading cause of global mortality, with malignant cells typically originating in the bladder’s urothelial lining. Urothelial bladder cancer (UBC) is the seventh most common cancer in men worldwide and ranks seventeenth in women^[1]^. Men are three to four times more likely than women to develop bladder cancer^[2]^. Early detection and intervention are crucial, yielding a 90% overall survival rate. Approximately, 75% of newly diagnosed UBC cases are non-invasive, classified as non-muscle invasive bladder cancer (NMIBC), with around 80% of initial bladder tumors falling under this category. NMIBC is primarily treated through transurethral resection of the bladder tumor (TURBT), followed by risk-based adjuvant intravesical therapy (IVe)^[3]^. For over 40 years, platinum-based chemotherapy has been the cornerstone of systemic treatment for muscle-invasive and advanced bladder cancer^[4]^. For intermediate and high-risk NMIBC patients, bacillus Calmette-Guérin (BCG) therapy administered intravesically after TURBT is the standard treatment. However, up to 40% of patients fail to respond to BCG and around 50% experience recurrence after an initial response.

## Current treatment options

The Food and Drug Administration (FDA) has approved various treatments for BCG-unresponsive NMIBC, including pembrolizumab, nadofaragene, valrubicin, and a combination of gemcitabine and docetaxel. However, a single, widely accepted standard of care for this condition has not yet been established. Radical cystectomy remains a viable treatment option for these patients, and participation in clinical trials is also encouraged^[5]^. While pembrolizumab is an effective therapy, it can lead to severe immune-related side effects, such as colitis, pneumonitis, and hepatitis, which may limit its use in elderly or more vulnerable patients. Nadofaragene, a gene therapy, requires specialized administration, making it less accessible. Additionally, the combination of gemcitabine and docetaxel is associated with significant side effects. Valrubicin is primarily used in patients with BCG-refractory carcinoma in situ (CIS) who cannot undergo surgery, which limits its application in a broader range of NMIBC cases. The existing data for these drugs are summarized in Table 1.

**Table 1:** Existing data for NMIBC drugs

Drug	Date of approval	Class	Mode of administration	Clinical trials
Pembrolizumab	January 8, 2020	PD-1 inhibitor	Intravenously every 3–6 weeks	Phase 2 clinical trial (NCT02625961)
Nadofaragene	December 16, 2022	IFNA2B gene therapies	Intravesical every 3 months	Phase 2 Clinical Trial (NCT01687244)
				Phase 3 Trial (Study 901, NCT02773849):
Valrubicin	June 30, 1998	Anthracycline	Intravesical once weekly for 6 weeks	Phase 2 Trial (NCT00003708)
				Phase 3 Clinical Trial (NCT00136373)
Gemcitabine	January 17, 2006	Antimetabolites	Intravenously on days 1 and 8 of a 21-day cycle	Phase 2 Study (NCT02202772)
				Phase 1/2 Study (NCT01619363)
Docetaxel	January 17, 2006	Taxanes	Intravenously on day 1 of a 21-day cycle	Phase 2 Study (NCT02202772)
				Phase 1/2 Study (NCT01619363)

## Anktiva: a new treatment option for NMBIC

On 22 April 2024, the FDA approved nogapendekin alfa inbakicept-pmln (Anktiva), an interleukin-15 (IL-15) receptor agonist. This approval covers its use, in combination with BCG, for treating adults with BCG-unresponsive NMIBC with CIS, with or without papillary tumors. Anktiva works by binding to IL-15 receptors on CD4+ and CD8+ T cells, as well as natural killer cells, stimulating their growth and activity while avoiding the suppression of regulatory T cells. In preclinical research, Anktiva demonstrated significant antitumor effects, both as a standalone therapy and when combined with BCG, showing better results than BCG alone in a carcinogen-induced bladder cancer model in immunocompetent rats^[6,7]^.

## Clinical trials

The FDA’s approval of Anktiva was based on results from the QUILT-3.032 clinical trial, which involved 77 adults diagnosed with high-risk, BCG-unresponsive NMIBC with CIS, with or without papillary tumors (PMID: 38226931). Prior to receiving a combination of Anktiva and BCG, all participants underwent transurethral resection of the tumor. The trial showed a complete response in 62% of patients, with 58% maintaining this response for 12 months or more. Additionally, 40% of patients sustained their response for at least 24 months, with some lasting up to 47 months. Importantly, no direct comparative studies have been conducted between Anktiva and other available treatments for NMIBC^[8]^. The 2021 guidelines from the European Association of Urology (EAU) traditionally recommended radical cystectomy as the standard approach for managing high-risk, BCG-unresponsive bladder tumors. However, utilizing pharmacological treatments such as Anktiva, rather than opting for surgical removal, is increasingly recognized as important for maintaining the patient’s quality of life and overall well-being^[9]^.

## Adverse effects

Clinical trials of Anktiva identified several adverse events, including elevated creatinine levels, dysuria, hematuria, frequent urination, urinary urgency, urinary tract infections, hyperkalemia, musculoskeletal pain, chills, and fever. Severe (Grade 3 or 4) cases occurred in less than 3% of patients for urinary tract infections, musculoskeletal pain, and hyperkalemia. Despite these side effects, no contraindications were found for Anktiva use^[6]^.

## Dosage

This medication is intended exclusively for intravesical administration. The recommended initial dose is 400 µg, given alongside BCG once a week for 6 weeks. If a complete response is not achieved by the third month, a second induction cycle may be considered. For ongoing maintenance, the same dosage is administered weekly for 3 weeks at months 4, 7, 10, 13, and 19. If a complete response is sustained by month 25 or later, additional maintenance doses can be given at months 25, 31, and 37. The drug comes in single-dose vials containing 400 µg/0.4 mL, which must be diluted and instilled within 2 hours of puncturing the vial^[6]^.

## Conclusion

The FDA’s approval of Anktiva offers a significant advancement for treating BCG-unresponsive NMIBC, particularly with CIS. Clinical trial results highlight its effectiveness, with a high rate of complete responses and sustained remissions. Anktiva’s unique mechanism of enhancing immune cell activity while sparing regulatory T cells positions it as a promising alternative to radical cystectomy, potentially improving patient outcomes and quality of life.

## Recommendations

This drug shows promise, but further research is needed to understand its effectiveness fully. While it’s available now, accessibility for everyone is crucial. Doctors should raise awareness about this drug and consider prescribing it when appropriate. Moreover, efforts should focus on reducing its cost to ensure widespread availability. Post-market evaluations are also essential to assess its performance and impact once it is in wider use.

## Data Availability

Not applicable.
